# Editors’ Book Table

**Published:** 1872-02

**Authors:** 


### Editors' Book Table.

[NOTE. — All works reviewed in the columns of the CHICAGO MEDICAL JOURNAL may be found in the extensive stock of W. B. KEEN, COOKE & Co whose catalogue of Medical Books will be sent to any address upon request.]

BOOKS RECEIVED.

*A Practical Treatise on the Diseases of Women.* By T. GAILLARD THOMAS, M.D., Professor of Obstetrics and Diseases of Women and Children in the College of Physicians and Surgeons, New York, etc., etc. Third edition, enlarged and thoroughly revised; with two hundred and forty-six illustrations on wood. Philadelphia: Henry C. Lea. 1872. Pp. 784.

The rapid exhaustion of previous editions attests the popularity of this work. And this popularity is most eminently merited. The text is lucid in exposition as it is sound and satisfactory in teaching. The wood-cut illustrations are not only numerous but excellent. It is as a whole by far the best American work on the subject, as it most certainly is superior to any foreign treatise which has been reproduced in this country. It is most cordially commended to our readers.

*Earth as a Topical Application in Surgery.* Being a full Exposition of its Use in all the Cases requiring Topical Applications admitted in the Men's and Women's Surgical Wards of the Pennsylvania Hospital during a period of Six Months in 1869. By ADDINELL HEWSON, M.D., one of the Attending Surgeons to the Pennsylvania Hospital;. with four Photo-Relief illustrations. "What relates to Truth is greater than what relates to Opinion."—*Bacon.* Philadelphia: Lindsay & Blakiston. 1872. Pp. 309.

A timely and instructive essay.

*When and How; Or a Collection of the more Recent Facts and Ideas upon Raising Healthy Children.* By DAN NEWCOMB, M.D. "An ounce of prevention is better than a pound of cure." Chicago: Arthur W. Penny & Co. 1872. Pp. 324.

A book rather for popular than professional use. So far as we have had opportunity to look it over we find its positions usually sound, and its style clear and forcible. Hygienic and not therapeutic.

PAMPHLETS RECEIVED.

*The Question of Quarantine: The Nature and Prevention of Communicable Zymotic Diseases.* A Paper read before the Medical Library and Journal Association of New York, Jan. 5, 1872. By ALFRED L. CARROLL, M.D., etc. From advance sheets of the "Medical Gazette." New York: F. Leypoldt, Publisher, 712 Broadway. 1872. Price fifty cents. Pp. 21.

A carefully considered monograph upon a very important topic. In summing up, the author calls attention to the following practical points:

"I. Communicable zymotic diseases depend upon material organic poisons, and although some of them, (as plague, cholera, etc.), may appear to be endemic in certain localities, it is probable that they exist there only by the retention and recrudescence of their specific contagion; it is almost certain, at all events, that they do not arise spontaneously elsewhere.

"II. 'Quarantine of observation' should in all instances apply to living beings in whom contagion may remain latent, rather than to inanimate substances which may be disinfected at once.

"III. Preventive measures should be adapted to the respective modes of contagion of the several disorders, stricter isolation being necessary in the case of those poisons which are volatile enough to be conveyed in the air, or in vapor of water, than with those which are transmissible only by solid or liquid media."

The discussion which followed the reading of Dr. Carroll's paper is particularly interesting, as is also Dr. Marsden's (of Montreal) plan for a Cholera Quarantine Station, which is given at length.

*Half-Yearly Compendium of Medical Science. Part IX. Jan.*, 1872. $3 per annum, single Nos. $2, Nos. 1 to 8 inclusive, $10. Philadelphia: S. W. Butler, M.D., 115 South Seventh Street.

